# Analysis of structural alterations in olfactory-related brain regions in patients with cognitive impairment and the correlation with olfactory function

**DOI:** 10.3389/fnagi.2026.1778960

**Published:** 2026-06-01

**Authors:** Xuefang Han, Xueshan Cao, Lin Liu, Ziqi Wang, Duo Gao, Huandi Lv, Haiqing Yang, Yiming Wang, Caixia Cui, Shenao Li, Qianhang Yang, Jingjing Cui, Zhengnan Zhang, Huixiao Wang, Shihan Cheng, Bo Song, Zuojun Geng

**Affiliations:** 1 Department of Medical Imaging, The Second Hospital of Hebei Medical University, Shijiazhuang, Hebei, China; 2 Department of Occupational Health and Environmental Health, School of Public Health, Hebei Medical University, Shijiazhuang, Hebei, China; 3 Key Laboratory of Environment and Human Health, Hebei Medical University, Shijiazhuang, Hebei, China; 4 Department of Research and Development, Shanghai United Imaging Intelligence, Co., Ltd, Shanghai, China; 5 Hebei Key Laboratory of Medical Imaging, Shijiazhuang, China

**Keywords:** Alzheimer’s disease, amygdala, hippocampus, magnetic resonance imaging, mild cognitive impairment, olfactory function

## Abstract

**Objective:**

To evaluate the differences in olfactory function, volumes of brain regions related to olfaction, and subregion volumes of the amygdala and hippocampus among the healthy control (HC), mild cognitive impairment (MCI), and Alzheimer’s disease (AD), as well as the association of volumes and olfactory function.

**Methods:**

Clinical data for the participants included age, gender, education level, Mini-Mental State Examination (MMSE). Participants underwent olfactory function test, including three sub-experiments as namely threshold test (TT), discrimination test (DT) and identification test (IT) and brain magnetic resonance imaging (MRI), including a three dimensions T1-weighted sequence (3D-T1WI). The brain subregions were extracted automatically by the uAI research portal.

**Results:**

A total of 107 participants were recruited, including 35 HC, 28 MCI and 44 AD. The volumes of hippocampal subregion (HC_Body_L and HC_Tail_L) were significant difference in HC and MCI groups while the global hippocampal volumes were not. All tested domains (TOS, TT, DT, and IT) showed statistically significant differences in olfactory function between the three groups. Furthermore, we found only IT showed significant differences across all three pairwise comparisons among the HC, MCI, and AD groups. The hippocampus and amygdala volumes showed the strongest correlation with olfactory function, followed by the PHG, PCC, lOFC and ERC, and more sub-region volumes had moderate-strength correlations with DT, IT, and TOS, according to Spearman correlation analysis.

**Conclusion:**

The olfactory function and the volumes of the hippocampal subnuclei maybe good candidates as early biomarkers of preclinical AD.

## 1 Introduction

With the sustained growth of economy and significant changes in social demographics, the issue of elderly-related diseases has increasingly drawn attention particularly. Alzheimer’s disease (AD), as a representative disease of neurodegenerative diseases has become a major challenge, affecting the health and quality of life among the elderly population severely. According to the “World Alzheimer’s Disease Report 2018” published by the Alzheimer’s Disease International (ADI), there are at least 50 million people with dementia worldwide, and it is projected to reach 152 million by 2050, with about 60–70 percent of them suffering from Alzheimer’s disease.

AD is a severe neurodegenerative disorder with complex etiology. Currently, due to the lack of effective treatment options, prevention of AD can be accomplished only by retarding the progression of the disease in its presymptomatic stages, thus postponing the onset of clinical symptoms (McDade et al., 2021). China is among the countries with the largest and fastest-growing elderly population globally, and identified as a high-risk nation for AD. According to data released by the Ministry of Civil Affairs at the end of 2022, China’s population aged 60 and above reached 280.04 million, accounting for 19.8% of the total population, and those aged 65 and above numbered 209.78 million, representing 14.9% (Zhi et al., 2025). In 2021, the number of people with Alzheimer’s disease and related dementias (ADRD) in China reached 16.99 million cases (Zhi et al., 2025). At the same time, the economic burden brought by Alzheimer’s disease is equally heavy. Total payments in 2023 for all individuals with Alzheimer’s or other dementias are estimated at $345 billion (Alzheimer’s and Dementia: The Journal of the Alzheimer’s Association, 2023). This shows that AD is a serious threat to the health and quality of life of the elderly, while also brings a heavy burden to families and society. Consequently, early prevention and diagnosis of AD emerges as one of the most crucial endeavors in the field of healthcare today.

Imaging markers based on magnetic resonance imaging (MRI) has a special foreground due to its wide coverage, ease of measurement and non-invasive nature. Especially the structural MRI (sMRI), which based on T1 Weighted Image can assess the brain atrophy being used as an image marker of neurodegenerative changes in brain aging (Aging Biomarker Consortium et al., 2023) and AD (Cummings, 2019). Jobin et al. ‘s research found that the total gray matter volume in the central olfactory processing region of the MCI group was significantly smaller than that of the healthy control group and the SCD group. Especially in the regions such as the piriform cortex, amygdala, entorhinal cortex, and hippocampus. More importantly, this atrophy is specific, and there is no significant difference in the volume of gray matter in non-olfactory areas among the three groups (Jobin et al., 2023). Papadatos et al. also demonstrated that there is a specific association between olfactory function and structural indicators of the olfactory brain region. In the SCD group, olfaction was associated with atrophy of the entorhinal cortex. In the MCI group, olfaction was related to hippocampal volume and the thickness of the right entorhinal cortex (Papadatos and Phillips, 2023).

Besides, previous studies also have shown that olfactory impairment is correlated with the pathological changes of AD (Doty, 2017; Lafaille-Magnan et al., 2017). Olfactory dysfunction gradually worsens with age (Murphy, 2002), and it is present in 85–90% of patients with AD, preceding the cognitive symptoms onset by several years (Schubert et al., 2008; Woodward et al., 2017). In the AD continuum, the onset of olfactory dysfunction begins as early as the subjective cognitive decline (SCD) stage, precedes brain structural changes, cognitive decline and clinical symptoms. The research also shows that the individuals with SCD had poorer olfactory function than healthy elderly, but better than MCI patients (Wang et al., 2021). This indicates that olfactory impairment may be a clinical marker, for the early detection and clinical diagnosis of AD.

At present, there are same reports on the correlation between olfactory function and olfactory related brain regions, but they are all based on individual brain regions. Comprehensive evaluations of olfactory related brain regions, such as amygdala (AMG), hippocampus (HPC), posterior cingulate cortex (PCC), insula (Ins), superior temporal gyrus (STG), entorhinal cortex (ERC), para-hippocampal gyrus (PHG), medial orbitofrontal cortex (mOFC), lateral orbitofrontal cortex (lOFC), have not been seen yet, and studies based on amygdala subregions and hippocampus subregions are even rare. Therefore, this study focuses on olfactory related brain regions as the target to evaluate the differences in olfactory function, brain regions volume, and subregion volumes of the amygdala and hippocampus among the three groups: healthy control (HC), mild cognitive impairment (MCI), and Alzheimer’s disease (AD). Additionally, the study explores the correlation between olfactory function and brain region volumes.

## 2 Materials and methods

### 2.1 Participants

The project adheres to the Declaration of Helsinki and has been approved by the Ethics Committee of the Second Hospital of Hebei Medical University. This study was conducted from March 2024 to September 2025 at the Second Hospital of Hebei Medical University. It was diagnosed as HC, MCI or AD by senior neurologists based on the National Institute on Aging and the Alzheimer’s Association (NIA-AA) criteria (2011) (Albert et al., 2011). Exclusion criteria for all participants were a history of psychiatric disorders, cardiovascular or cerebrovascular disease, nasal pathologies, alcohol or substance abuse, steroid treatment, infections, inability to undergo clinical tests or MRI scanning, presence of obvious head motion during scanning or other reasons causing image artifacts. A total of 107 participants were recruited, including 35 HC, 28 MCI and 44 AD, collected demographic information including age, gender, and education level for all participants. All the participants signed the informed consent form. All participants were right-handed and aged between 53 and 81 years.

### 2.2 Olfactory function test

Olfactory function testing was performed using Humans’ Quick Odour Pens “Sniffin’ Sticks” in a quiet and well-ventilated indoor place, which included three sub-experiments, namely threshold test (TT), discrimination test (DT) and identification test (IT). Odor threshold is the ability to detect an odor at a given concentration wherein the lowest detectable concentration is considered the threshold; Odor discrimination is the ability to distinguish between two or more odors; Odor identification is the detection and recall of a previous smell associated with an individual’s knowledge or experience.

During smell administration, the opened odour pen was set at a distance about 2 cm in front of both nostrils for about 3–4 s. The interval between the different presentations should be around 30 s. A break of about 3 min should be made between the three tests. The complete test took 30–40 min. The olfactory function test result was expressed as the sum of the three sub-test scores. A total score of more than 30 was normal, and a total score of less than 30 indicated the decreased sense of smell.

### 2.3 MRI data acquisition

Brain MRI data were acquired using a GE Architect 3.0 T MR-scanner with a 48-channel phased-array head coil. Structural images were acquired with high-resolution three dimensions T1- weighted fast field echo structural scans (repetition time 6.2 ms, echo time 2.4 ms, field of view 256 mm × 256 mm, flip angle 8°, and voxel size 1 mm × 1 mm × 1 mm).

The brain subregions of all groups were extracted automatically by the uAI research portal (uRP, https://urp.united-imaging.com). The uRP is a multifunctional platform to perform accurate image processing and analysis (Wu et al., 2023). The processing pipeline involved automatic parcellation of the structural MRI data using a 3D deep learning segmentation model, which achieved a Dice similarity coefficient of 91.06% between the automatically segmented results and ground truth data. Note that the ground truth is initially obtained from FreeSurfer software and then further refined by an experienced rater based on the Desikan–Killiany atlas (Desikan et al., 2006; Fischl, 2012) The image processing flow mainly includes: (a) the bias field correction, (b) removal of the skull, (c) tissue segmentation of white matter, gray matter, and cerebrospinal fluid (CSF), (d) bilateral segmentation, and (e) parcellation of 109 sub-regions. Following the brain parcellation step, brain volumes were automatically calculated.

Different olfactory functions are regulated by different regions of the brain which are also relevant to aging, PD and AD (Dan et al., 2021). The key brain regions in humans and mammalian animal models involved in olfactory function are the entorhinal cortex, hippocampus, insula, orbitofrontal cortex, inferior frontal gyrus, piriform cortex, thalamus, cingulate gyrus, and amygdala (Kehl et al., 2024; Stenwall et al., 2025). Retrospectively, odor identification were associated with the brain volumes of specific frontal (medial frontal, orbitofrontal, insula, and precentral) and temporal areas (middle, superior, inferior, entorhinal cortex, parahippocampal, amygdala, fusiform, and hippocampus), and thalamus (Tian et al., 2023). Based on the above, the following brain regions were selected: intracranial volume (ICV) of the subjects (sum of gray matter, white matter and intracranial cerebrospinal fluid volume), and brain regions related to olfaction including AMG, HPC, PCC, Ins, STG, ERC, PHG, mOFC and lOFC.

The AMG and HPC play a central role in olfactory information processing and odor identification (Kehl et al., 2024). Therefore, we further conducted subregion analysis of the amygdala and hippocampus. The amygdala subregions include lateral nucleus (La), basal nucleus (Ba), central nucleus (Ce), medial nucleus (Me), cortical nucleus (Co), accessory basal nucleus (AB), cortico-amygdaloid transition area (CAT), anterior amygdaloid area (AAA), paralaminar nucleus (PL). The hippocampal subregions includes hippocampal head (HC_Head), hippocampal body (HC_Body), hippocampal tail (HC_Tail), hippocampal parasubiculum (HC_Parasubiculum), hippocampal presubiculum (HC_Presubiculum), hippocampal subiculum (HC_Subiculum), HC_CA1, HC_CA3, HC_CA4, hippocampal granule cell layer of dentate gyrus (HC_GC_DG), hippocampal molecular layer (HC_ Molecular layer), hippocampus amygdala transition area (HC_HATA), hippocampal fimbria (HC_Fimbria), hippocampal fissure (HC_Fissure).

### 2.4 Statistical analysis

Statistical analysis was performed in SPSS 31.0 software (IBM, Armonk, NY, United States) and R Studio. Data with normal distribution are expressed as mean ± standard deviation (SD), and non-normal distribution are expressed as median (interquartile range). For numerical data, such as age and MMSE, One-way ANOVA or a Kruskal-Wallis test was used to evaluate the differences among HC, MCI and AD groups. For categorical data, such as gender and education, Pearson’s Chi-squared test was used to evaluate differences among HC, MCI and AD groups. Comparisons of olfactory function among the HC, MCI, and AD groups were performed using ANCOVA, with age, gender, and education included as covariates. Comparisons of brain regional and subregional volumes among the HC, MCI, and AD groups were performed using ANCOVA, with age, gender, education, and ICV included as covariates. Post-hoc comparisons were corrected for multiple testing using Bonferroni correction. A Spearman partial correlation analysis was used to evaluate the correlations between olfactory function and brain regional volumes by controlling for age, gender, education and ICV. Statistical significance was considered as *p* < 0.05.

## 3 Results

### 3.1 Basic characteristics of the participants

**Figure 1:**
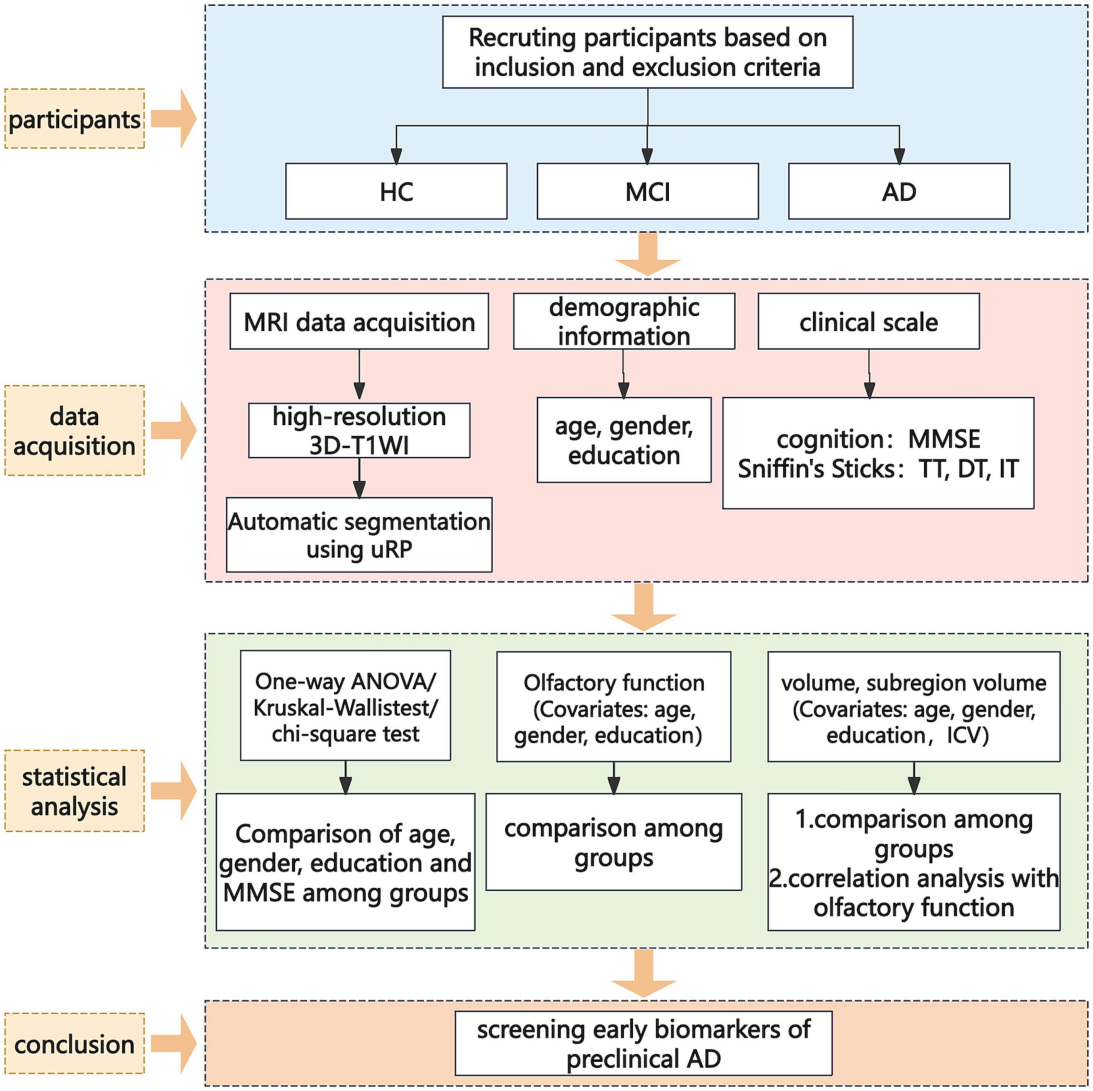
Study’s flow diagram.

Figure 1 displays the study’s flow diagram. A total of 35 HC participants, 28 MCI patients and 44 AD patients with high-resolution sMRI data were enrolled. The mean age for the cohort was 66.2 ± 6.5 years. The demographic data for participants by group are shown in Table 1. No significant age or gender differences were observed between the HC, MCI and AD groups (*p* > 0.05). There were significant differences between the three groups in terms of education attainment and MMSE scores.

**Table 1:** Statistical analysis of demographic information in groups HC, MCI, and AD.

Index	HC (*n* = 35)	MCI (*n* = 28)	AD (*n* = 44)	*H*/*χ*^2^	*P*	*P*1	*P*2	*P*3
Age (years)	63 (61, 67)	66.11 ± 7.12	67.70 ± 7.42	5.682	0.058	–	–	–
Gender (male/female)	19/16	15/13	22/22	0.167	0.920	–	–	–
Education								
Primary school and below	1	3	10	15.135	<0.001	0.105	<0.001	0.054
Junior high school	6	10	20					
Senior high school	18	8	8					
University and above	10	7	6					
MMSE score	29 (28, 30)	25 (25, 26.75)	16.5(12, 21)	89.788	<0.001	0.001	<0.001	<0.001

HC, healthy control; MCI, mild cognitive impairment; AD, Alzheimer’s disease; MMSE, Mini-Mental State Examination.

*p* values for comparison among the three groups.

*P*1 HC group versus MCI group.

*P*2 HC group versus AD group.

*P*3 MCI group versus AD group.

### 3.2 Olfactory function

Table 2 displays the findings of the measurement and statistical analysis of the total olfactory score (TOS), TT, DT and IT scores. All tested domains (TOS, TT, DT, and IT) showed statistically significant differences in olfactory function between the three groups. We also observed that olfactory function declined with disease progression. With the exception of the comparison in the TT between the gropes of MCI and AD (*p* = 0.991), the DT between the gropes of HC and MCI (*p* = 0.137) and the TOS between the gropes of MCI and AD (*p* = 0.101), *post hoc* analysis using the Dunn Kruskal-Wallis multiple comparison (*p*-values adjusted with the Bonferroni method) or LSD method revealed significant differences in olfactory function among all other inter-group comparisons (*p* < 0.05).

**Table 2:** Statistical analysis of the olfactory function in groups HC, MCI, and AD.

Index	HC (*n* = 35)	MCI (*n* = 28)	AD (*n* = 44)	*F*/*W*	*p*	*P*1	*P*2	*P*3
Olfactory function								
Threshold test	7.848 ± 0.621	4.935 ± 0.581	4.926 ± 0.546	14.225	<0.001	0.002	0.002	0.991
Discrimination test	10.122 ± 0.424	9.198 ± 0.430	7.159 ± 0.465	23.037	<0.001	0.137	<0.001	0.002
Identification test	10.795 ± 0.526	7.449 ± 0.559	5.615 ± 0.469	25.014	<0.001	<0.001	<0.001	0.042
TOS	28.783 ± 1.292	21.600 ± 1.374	17.718 ± 1.152	18.935	<0.001	<0.001	<0.001	0.101

*p* values for comparison among the three groups.

*P*1 HC group versus MCI group.

*P*2 HC group versus AD group.

*P*3 MCI group versus AD group.

### 3.3 Brain regions volumes

Table 3 displays the findings of the measurement and statistical analysis of the ICV volumes and the olfactory related brain regions. Except for ICV, the volumes of the other olfactory related brain regions all showed intergroup differences between the three groups. Further pairwise comparisons revealed that there were significantly differences between the AD group and the MCI group as well as between the AD group and the HC group in the volumes of both the left and right of AMG, HPC, PCC, Ins, STG, ERC, PHG and lOFC as well as the right side of mOFC. There were no differences between the AD and MCI groups in the left side of mOFC or between the HC and MCI groups in any of the regions.

**Table 3:** Statistical analysis of the ICV volumes and the olfactory related brain regions.

MRI measures	HC (*n* = 35)	MCI (*n* = 28)	AD (*n* = 44)	*F*/*W*	*p*	*P*1	*P*2	*P*3
ICV	1,430.237 ± 116.272	1,415.199 ± 115.811	1,395.674 ± 122.754	0.837	0.436	–	–	–
AMG_L	2.046 ± 0.061	1.991 ± 0.063	1.507 ± 0.040	69.201	<0.001	0.519	<0.001	<0.001
AMG_R	2.086 ± 0.052	2.022 ± 0.056	1.527 ± 0.047	35.410	<0.001	1.000	<0.001	<0.001
HPC_L	3.352 ± 0.073	3.152 ± 0.077	2.486 ± 0.065	40.149	<0.001	0.183	<0.001	<0.001
HPC_R	3.445 ± 0.070	3.262 ± 0.075	2.638 ± 0.063	37.635	<0.001	0.227	<0.001	<0.001
PCC_L	3.073 ± 0.080	3.014 ± 0.064	2.618 ± 0.061	27.491	<0.001	0.565	<0.001	<0.001
PCC_R	3.129 ± 0.073	3.052 ± 0.077	2.643 ± 0.065	13.426	<0.001	1.000	<0.001	<0.001
Ins_L	6.712 ± 0.099	6.733 ± 0.105	6.230 ± 0.089	8.574	<0.001	1.000	0.002	0.001
Ins-R	6.834 ± 0.100	6.740 ± 0.107	6.310 ± 0.090	8.025	<0.001	1.000	0.001	0.008
STG_L	11.374 ± 0.201	11.432 ± 0.213	10.639 ± 0.180	5.070	0.008	1.000	0.032	0.017
STG_R	10.777 ± 0.177	10.749 ± 0.186	10.024 ± 0.146	13.288	0.001	0.912	0.005	0.005
ERC_L	2.075 ± 0.067	1.994 ± 0.068	1.637 ± 0.047	33.044	<0.001	0.393	<0.001	<0.001
ERC_R	1.951 ± 0.064	1.878 ± 0.065	1.571 ± 0.046	26.555	<0.001	0.425	<0.001	<0.001
PHG_L	2.148 ± 0.042	2.044 ± 0.042	1.728 ± 0.030	70.726	<0.001	0.079	<0.001	<0.001
PHG_R	1.993 ± 0.040	1.942 ± 0.042	1.643 ± 0.036	23.324	<0.001	1.000	<0.001	<0.001
lOFC_L	6.726 ± 0.124	6.638 ± 0.129	6.087 ± 0.010	18.345	<0.001	0.621	<0.001	0.002
lOFC_R	6.809 ± 0.091	6.756 ± 0.096	6.373 ± 0.081	7.154	0.001	1.000	0.003	0.009
mOFC_L	5.038 ± 0.079	4.924 ± 0.083	4.688 ± 0.070	5.290	0.007	0.967	0.006	0.101
mOFC_R	5.291 ± 0.080	5.148 ± 0.082	4.795 ± 0.064	23.541	<0.001	0.207	<0.001	0.002

*p* values for comparison among the three groups.

*P*1 HC group versus MCI group.

*P*2 HC group versus AD group.

*P*3 MCI group versus AD group.

Table 4 displays the findings of the measurement and statistical analysis of the volumes of the 18 subregions in the left and right amygdalae. Excepted Me_L and PL_L volumes between the HC and AD groups and Me_L, Me_R and PL_L volumes between the MCI and AD groups all other subregion volumes demonstrated significant differences between the HC and AD groups as well as between the MCI and AD groups.

**Table 4:** Statistical analysis of volumes in 18 subregions of the left and right amygdala.

MRI measures	HC (*n* = 35)	MCI (*n* = 28)	AD (*n* = 44)	*F*/*W*	*p*	*P*1	*P*2	*P*3
La_L	0.641 ± 0.019	0.643 ± 0.021	0.496 ± 0.013	51.085	<0.001	0.943	<0.001	<0.001
La_R	0.640 ± 0.021	0.646 ± 0.023	0.497 ± 0.015	43.229	<0.001	0.848	<0.001	<0.001
Ba_L	0.451 ± 0.014	0.443 ± 0.014	0.334 ± 0.009	66.722	<0.001	0.687	<0.001	<0.001
Ba_R	0.468 ± 0.015	0.455 ± 0.015	0.346 ± 0.010	59.798	<0.001	0.536	<0.001	<0.001
Ce_L	0.034 ± 0.001	0.031 ± 0.001	0.022 ± 0.001	61.650	<0.001	0.068	<0.001	<0.001
Ce_R	0.035 ± 0.001	0.032 ± 0.001	0.023 ± 0.001	34.393	<0.001	0.148	<0.001	<0.001
Me_L	0.002 ± 0.0002	0.002 ± 0.0002	0.002 ± 0.0002	2.421	0.298	–	–	–
Me_R	0.003 ± 0.000	0.002 ± 0.000	0.002 ± 0.000	3.181	0.046	0.712	0.040	0.584
Co_L	0.021 ± 0.001	0.019 ± 0.001	0.015 ± 0.000	45.413	<0.001	0.168	<0.001	<0.001
Co_R	0.021 ± 0.001	0.019 ± 0.001	0.015 ± 0.001	25.413	<0.001	0.265	<0.001	<0.001
AB_L	0.258 ± 0.009	0.247 ± 0.009	0.182 ± 0.005	68.156	<0.001	0.378	<0.001	<0.001
AB_R	0.264 ± 0.009	0.252 ± 0.009	0.184 ± 0.006	69.774	<0.001	0.336	<0.001	<0.001
CAT_L	0.150 ± 0.005	0.145 ± 0.005	0.108 ± 0.003	70.969	<0.001	0.384	<0.001	<0.001
CAT_R	0.154 ± 0.005	0.147 ± 0.005	0.108 ± 0.003	64.565	<0.001	0.341	<0.001	<0.001
AAA_L	0.025 ± 0.001	0.024 ± 0.001	0.017 ± 0.001	62.659	<0.001	0.302	<0.001	<0.001
AAA_R	0.027 ± 0.001	0.025 ± 0.001	0.018 ± 0.001	75.653	<0.001	0.153	<0.001	<0.001
PL_L	0.031 ± 0.001	0.032 ± 0.001	0.028 ± 0.001	3.049	0.052	–	–	–
PL_R	0.036 ± 0.001	0.036 ± 0.001	0.027 ± 0.001	36.924	<0.001	0.953	<0.001	<0.001

*p* values for comparison among the three groups.

*P*1 HC group versus MCI group.

*P*2 HC group versus AD group.

*P*3 MCI group versus AD group.

Table 5 displays the findings of the measurement and statistical analysis of the volumes of the 28 subregions in the left and right hippocampal. There were no statistically significant differences in the volumes of HATA_L, HATA_R and HC_Fissure_R across the HC, MCI, and AD groups. There were statistically significant differences between the HC and MCI groups only in the volumes of the HC_Body_L and HC_Tail_L subregions. All other subregion volumes showed significant differences between the HC and AD groups, as well as between the MCI and AD groups, excepted of HC_Presubiculum_R and HC_ HC_Fissure_L volumes between the HC and AD groups.

**Table 5:** Statistical analysis of volumes in 28 subregions of the left and right hippocampal.

MRI measures	HC (*n* = 35)	MCI (*n* = 28)	AD (*n* = 44)	*F*/*W*	*p*	*P*1	*P*2	*P*3
HC_Head_L	1.607 ± 0.040	1.558 ± 0.042	1.189 ± 0.035	34.655	<0.001	1.000	<0.001	<0.001
HC_Head_R	1.688 ± 0.039	1.617 ± 0.041	1.278 ± 0.035	33.176	<0.001	0.633	<0.001	<0.001
HC_Body_L	1.148 ± 0.021	1.079 ± 0.030	0.835 ± 0.027	70.131	<0.001	0.047	<0.001	<0.001
HC_Body_R	1.154 ± 0.027	1.106 ± 0.029	0.864 ± 0.024	34.148	<0.001	0.672	<0.001	<0.001
HC_Tail_L	0.538 ± 0.013	0.479 ± 0.017	0.376 ± 0.157	50.717	<0.001	0.005	<0.001	<0.001
HC_Tail_R	0.543 ± 0.016	0.491 ± 0.017	0.388 ± 0.015	23.993	<0.001	0.093	<0.001	<0.001
HC_Parasubiculum_L	0.038 ± 0.002	0.038 ± 0.002	0.026 ± 0.002	10.410	<0.001	1.000	<0.001	<0.001
HC_Parasubiculum_R	0.036 ± 0.003	0.035 ± 0.003	0.024 ± 0.002	14.406	<0.001	0.759	0.004	0.009
HC_Presubiculum_L	0.322 ± 0.009	0.308 ± 0.010	0.217 ± 0.008	38.951	<0.001	0.907	<0.001	<0.001
HC_Presubiculum_R	0.288 ± 0.009	0.282 ± 0.010	0.207 ± 0.008	26.382	<0.001	1.000	0.050	0.045
HC_Subiculum_L	0.418 ± 0.013	0.395 ± 0.013	0.295 ± 0.008	72.578	<0.001	0.216	<0.001	<0.001
HC_Subiculum_R	0.433 ± 0.010	0.407 ± 0.011	0.312 ± 0.009	42.296	<0.001	0.217	<0.001	<0.001
HC_CA1_L	0.644 ± 0.014	0.621 ± 0.022	0.484 ± 0.017	48.602	<0.001	0.356	<0.001	<0.001
HC_CA1_R	0.703 ± 0.020	0.666 ± 0.020	0.546 ± 0.014	44.932	<0.001	0.199	<0.001	<0.001
HC_CA3_L	0.208 ± 0.005	0.193 ± 0.008	0.157 ± 0.007	29.955	<0.001	0.127	<0.001	0.001
HC_CA3_R	0.218 ± 0.007	0.213 ± 0.007	0.166 ± 0.006	18.768	<0.001	1.000	<0.001	<0.001
HC_CA4_L	0.233 ± 0.005	0.225 ± 0.005	0.187 ± 0.005	23.093	<0.001	0.802	<0.001	<0.001
HC_CA4_R	0.246 ± 0.004	0.236 ± 0.006	0.196 ± 0.006	40.081	<0.001	0.167	<0.001	<0.001
HC_GC_DG_L	0.278 ± 0.006	0.273 ± 0.007	0.223 ± 0.006	22.598	<0.001	1.000	<0.001	<0.001
HC_GC_DG_R	0.292 ± 0.008	0.282 ± 0.008	0.234 ± 0.006	37.830	<0.001	0.362	<0.001	<0.001
HC_Molecular layer_L	0.527 ± 0.009	0.500 ± 0.016	0.377 ± 0.012	83.950	<0.001	0.127	<0.001	<0.001
HC_Molecular layer_R	0.552 ± 0.013	0.529 ± 0.013	0.408 ± 0.011	38.320	<0.001	0.696	<0.001	<0.001
HC_HATA_L	0.015 ± 0.002	0.014 ± 0.002	0.011 ± 0.002	1.644	0.440	–	–	–
HC_HATA_R	0.016 ± 0.002	0.012 ± 0.002	0.012 ± 0.002	1.916	0.384	–	–	–
HC_Fimbria_L	0.070 ± 0.004	0.062 ± 0.004	0.043 ± 0.003	14.095	<0.001	0.422	<0.001	0.001
HC_Fimbria_R	0.054 ± 0.004	0.055 ± 0.004	0.034 ± 0.003	10.684	<0.001	1.000	<0.001	<0.001
HC_Fissure_L	0.090 ± 0.005	0.074 ± 0.005	0.094 ± 0.004	4.584	0.012	0.085	1.000	0.014
HC_Fissure_R	0.103 ± 0.007	0.095 ± 0.006	0.112 ± 0.006	3.897	0.142	–	–	–

*p* values for comparison among the three groups.

*P*1 HC group versus MCI group.

*P*2 HC group versus AD group.

*P*3 MCI group versus AD group.

Furthermore, we observed that most volumes of olfactory-related brain regions, amygdala subregions, and hippocampal subregions decreased with decreasing cognitive function. The volumes of Ins_L, STG_L, La_L, La_R, PL_L, and HC_Fimbria_R were slightly larger in the MCI group than in the HC group, but there were no significant differences (*p* > 0.05).

### 3.4 Correlation between olfactory function and brain region volume

**Figure 2:**
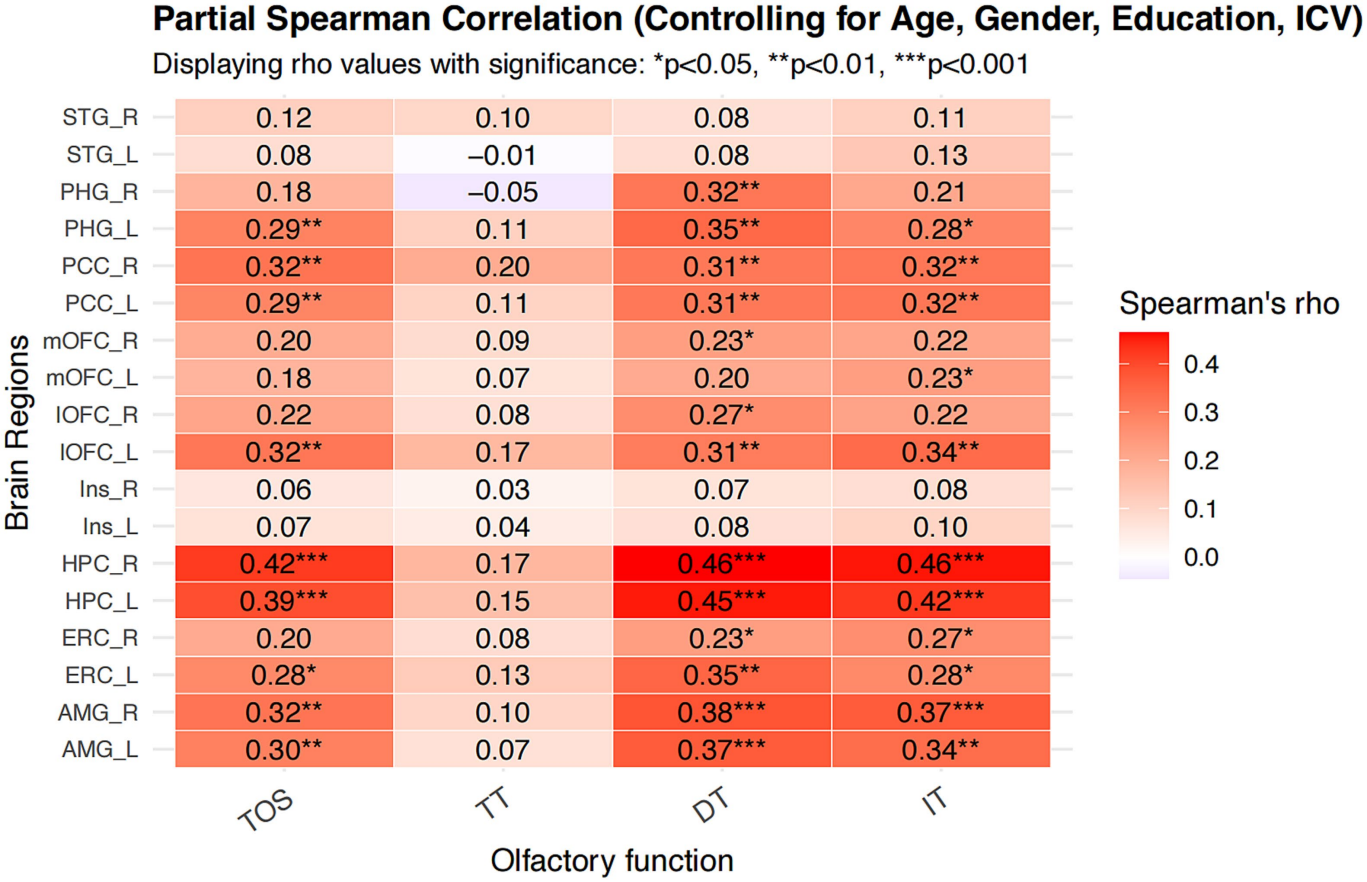
Correlation analysis between olfactory function and the olfactory related brain region volumes.

The correlations between olfactory function and brain region volumes were examined using Spearman partial correlation analysis, controlling for age, gender, education, and ICV as covariates. The outcomes are shown in Figure 2.

The figure shows that all nine brain regions—aside from STG_L and PHG_R—have a positive correlation with olfaction function. The relationship between TT and volumes is rather weak. Furthermore, the DT, IT and TOS all have relatively strongest correlation with HPC_R (*r* = 0.46, 0.46, 0.42), HPC_L (*r* = 0.45, 0.42, 0.39), AMG_R (*r* = 0.38, 0.37, 0.32) and AMG_L (*r* = 0.37, 0.34, 0.30). Besides, PHG_L, PCC_L, PCC_R, lOFC_L and ERC_L also exhibit a certain degree of correlation with DT, IT, and TOS.

**Figure 3:**
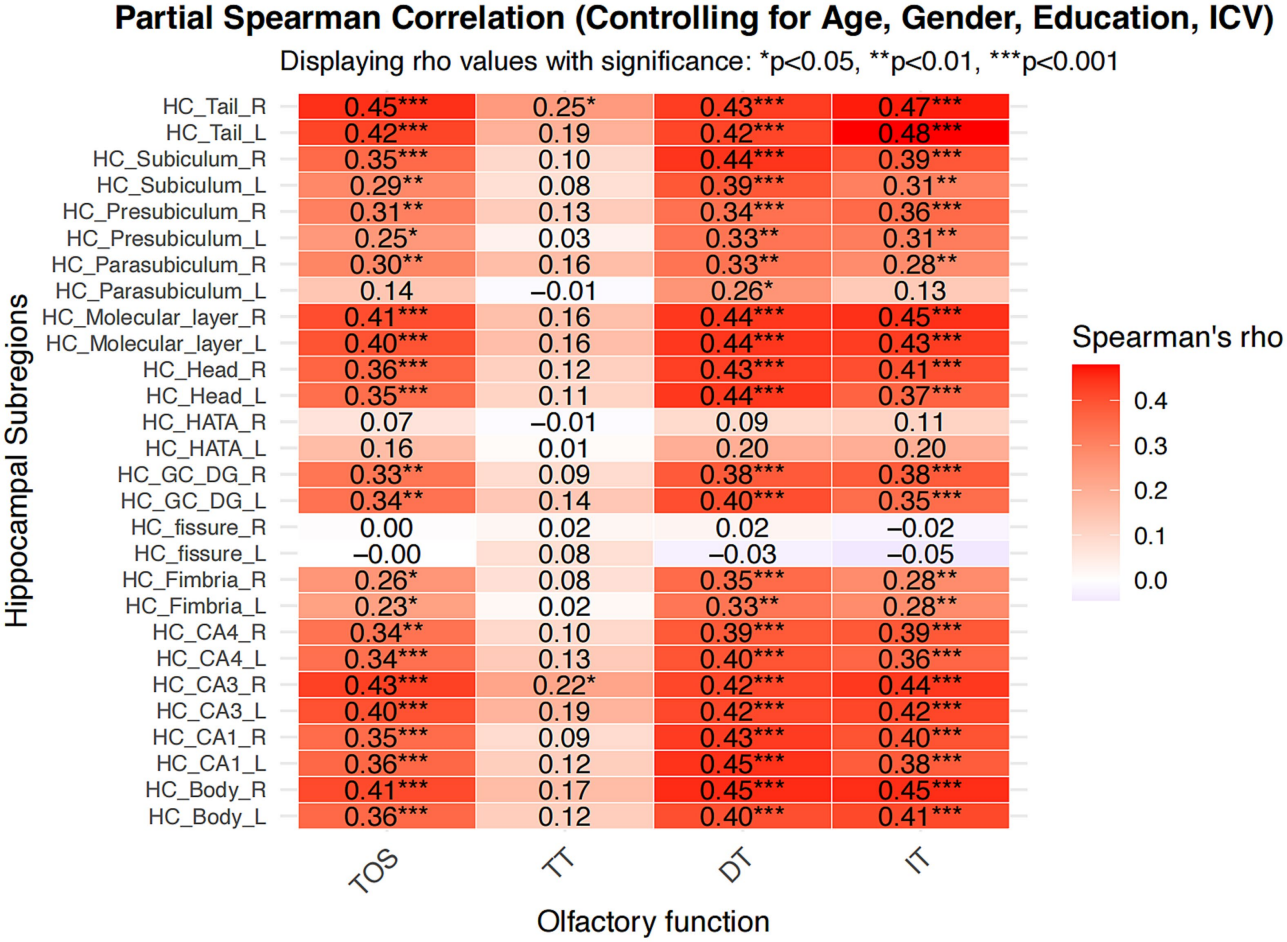
Correlation analysis between hippocampal subregion volumes and olfactory function.

Figure 3 displays the correlation analysis between olfactory function and hippocampal subregion volumes. Olfactory threshold, odor discrimination ability, or identification ability do not appear to be correlated with HC_Fissure. Only the HC_tail_R (*r* = 0.25) and HC_CA3_R (*r* = 0.22) showed a weak correlation with olfactory thresholds, according to the results of the correlation between hippocampal subregion volumes and olfactory threshold. Brain regions within the hippocampal subregion exhibiting moderate-strength correlations with DT include HC_Tail_R (*r* = 0.43), HC_Tail_L (*r* = 0.42), HC_Subiculum_R (*r* = 0.44), HC_Molecular_layer_R (*r* = 0.44), HC_Molecular_ layer_L (*r* = 0.44), HC_Head_R (*r* = 0.43), HC_Head_L (*r* = 0.44), HC_GC_DG_L (*r* = 0.40), HC_CA4_L (*r* = 0.4), HC_CA3_R (*r* = 0.42), HC_CA3_L (*r* = 0.42), HC_CA1_R (*r* = 0.43), HC_CA1_L (*r* = 0.45), HC_Body_R (*r* = 0.45), HC_Body_L (*r* = 0.40). Brain regions within the hippocampal subregion exhibiting moderate-strength correlations with IT include HC_Tail_R (*r* = 0.47), HC_Tail_L (*r* = 0.48), HC_Molecular_layer_R (*r* = 0.45), HC_Molecular_ layer_L (*r* = 0.43), HC_Head_R (*r* = 0.41), HC_CA3_R (*r* = 0.44), HC_CA3_L (*r* = 0.42), HC_CA1_R (*r* = 0.40), HC_Body_R (*r* = 0.45), HC_Body_L (*r* = 0.41). Brain regions within the hippocampal subregion exhibiting moderate-strength correlations with TOS include HC_Tail_R (*r* = 0.45), HC_Tail_L (*r* = 0.42), HC_Molecular_layer_R (*r* = 0.41), HC_Molecular_ layer_L (*r* = 0.40), HC_CA3_R (*r* = 0.43), HC_CA3_L (*r* = 0.40), HC_Body_R (*r* = 0.41).

**Figure 4:**
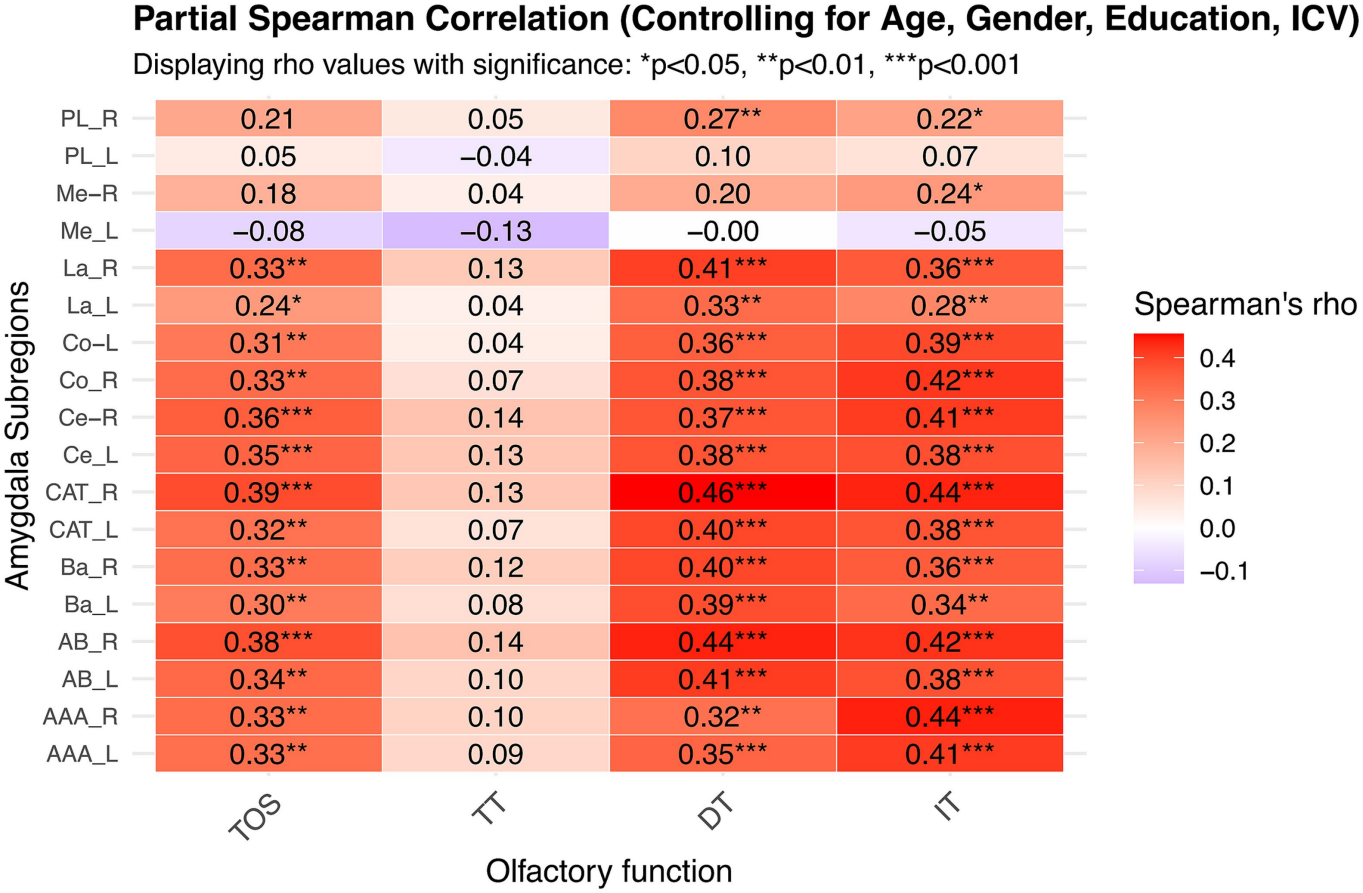
Correlation analysis between amygdala subregion volumes and olfactory function.

Figure 4 displays the correlation analysis between olfactory function and the volumes of the amygdala subregion. The amygdala subregion volumes had a weaker correlation with TT and were not statistically significant (*p* > 0.05). Brain regions within the amygdala subregion exhibiting moderate-strength correlations with DT include LA_R (*r* = 0.41), CTA_R (*r* = 0.46), CTA_L (*r* = 0.40), BA_R (*r* = 0.40), AB_R (*r* = 0.44), AB_L (*r* = 0.41). Brain regions within the amygdala subregion exhibiting moderate-strength correlations with IT include CO_R (*r* = 0.42), CE_R (*r* = 0.41), CTA_R (*r* = 0.44), AB_R (*r* = 0.42), AAA_R (*r* = 0.44), AAA_L (*r* = 0.41). Multiple amygdala subregions exhibit weak volumetric correlations with TOS.

## 4 Discussion

The onset and progression of Alzheimer’s disease is a lengthy process characterized by significant individual variation, beginning with asymptomatic pathological changes in the brain, followed by increasing pathological burden, and leading to the emergence of clinical symptoms and continuous progression (Alzheimer’s and Dementia: The Journal of the Alzheimer’s Association, 2023). The pathological changes of AD begin to occur silently in the brain more than a decade before clinical symptoms appear. These changes first selectively affect specific brain regions, then spread along neuroanatomical networks, ultimately leading to widespread brain atrophy and irreversible cognitive decline. However, what has long troubled us is that AD remains incurable. Furthermore, because AD is often diagnosed late, interventional trials aimed at slowing its progression are frequently implemented too late to yield significant benefits (Mehta et al., 2017). If intervention or prevention were initiated before the onset of clinical symptoms, it might effectively slow the progression of the disease (McDade et al., 2021). Therefore, identifying effective biomarkers is crucial for the early recognition and diagnosis of AD and represents a current priority in scientific research.

In the current study, the main goal was to investigate changes in the olfactory function and the volumes of brain regions related to olfaction, in patients with AD and MCI compared with HC. Subregional analyses were conducted for brain regions closely related to cognitive function, such as the amygdala and hippocampus. It is anticipated that imaging biomarkers for the effective and early diagnosis of the Alzheimer’s disease continuum will be discovered.

Through analysis of demographic data, we found that the proportion of individuals with higher education was higher in the HC group than in both the MCI and AD groups. Just as the Lancet previously reported that higher education or continue cognitive activity would prevent and delay dementia by reducing dementia neuropathology, reducing stress and inflammation, building cognitive and brain reserve. Therefore the people with more childhood education and higher educational attainment have a reduced dementia risk (Livingston et al., 2024).

Olfactory dysfunction is common in AD and other neurodegenerative diseases (McLaren and Kawaja, 2024). This study further support the notion that olfactory function is related to cognitive impairment and compared to cognitively normal research participants, patients with cognitive abnormalities exhibited reduced olfactory function, as demonstrated in the study by Tian Q and Christina S. Dintica (Dintica et al., 2019; Tian et al., 2023).

As we all know, accumulation of pathological deposition of Amyloid-beta (A*β*), phosphorylated tau, and *α*-synuclein in the brain has been considered as markers of neurodegenerative diseases including Alzheimer’s, as well as physiological markers of brain aging (Mattsson et al., 2009; Silva and Haggarty, 2020; Zhang et al., 2021; Sengupta and Kayed, 2022; Leuzy et al., 2025). A Systematic Review and Meta-Analysis of several biological mechanisms related to the associations between olfactory identification ability and recognition found that olfactory identification ability was negatively correlated with Aβ PET and CSF total tau (Tu et al., 2020). These studies have also confirmed from a pathological perspective that the reduction of olfactory function is positively correlated with the decline in cognitive degree.

The neuropathological basis of olfactory dysfunction in AD is thought to be due mainly to accumulations of disease-related lesions, especially tau pathology in AD, that occur in the olfactory bulb and primary olfactory sensory cortices of the cerebrum (Arnold et al., 1991; Olfactory tau pathology in Alzheimer disease and mild cognitive impairment-PubMed, n.d.). PHFtau, α-synuclein and amyloid-β lesions occur early and severely in cerebral regions of the olfactory system and they have also been observed in olfactory epithelium (OE) (Arnold et al., 2010). Therefore, we hypothesize that the volume of olfactory-related brain regions will also be altered with the occurrence of cognitive impairment.

The ICV was no difference among the three groups. However, the results indicated that the volume of olfactory-related brain regions differed significantly among the three groups. The results of this study confirm that the volumes of AMG, HPC, PCC, Ins, STG, ERC, PHG, mOFC and lOFC were all reduced in the AD group compared with the MCI group and the HC group. The volumes of Ins_L, STG_L, La_L, La_R, PL_L, and HC_Fimbria_R were slightly larger in the MCI group than in the HC group, but there were no significant differences. According to existing literatures, many neuroimaging investigations have identified atrophy of the subcortical structure, as a distinctive feature of AD and MCI (Mijares Diaz et al., 2025). However, some studies have reported different findings. Specifically, Yang et al. observed surface area and local gyrification index in certain cortical regions were increased in the MCI group, such as the transverse temporal gyrus, superior temporal gyrus, and insula (Yang et al., 2023). These morphological changes may account for the relative increases in volumetric measures. We propose that this may result from structural remodeling or compensatory mechanisms during the early stages of the disease process. Olfactory functions, including TT, DT, IT, and TOS, gradually decreased over the course of disease progression, although some differences between groups were not statistically significant. Just as the research by Tian Q higher odor identification scores were associated with greater brain volumes mostly in specific areas, such as orbitofrontal, insula, middle, inferior, entorhinal cortex, amygdala, hippocampus, thalamus, posterior cingulate, corpus callosum, and occipital white matter (Tian et al., 2023). Furthermore, we found that only IT showed significant differences across all three pairwise comparisons among the HC, MCI, and AD groups, suggesting that IT has relatively stronger discriminative ability among the three groups. This finding is consistent with previous literature, which has reported that among the three aspects of olfactory function, odor identification exhibits the strongest correlation with the risk of dementia (Trapp et al., 2023; Xie et al., 2024). Our research findings add to the existing literature and further support the concept that olfaction function is related to cognitive impairment.

Through Spearman partial correlation analysis between brain regions volumes with olfactory function, we found that the volumes of the hippocampus and amygdala showed the strongest correlation with olfactory function and followed by the PHG, PCC, lOFC and ERC. This was in line with previous studies reported that higher odor identification scores were significantly associated with slower rates of brain atrophy in specific frontal (medial frontal, orbitofrontal, insula, and precentral) and temporal areas (middle, superior, inferior, entorhinal cortex, parahippocampal, amygdala, fusiform, and hippocampus) (Tian et al., 2023). Presence of AD neuropathological alterations such as large numbers of neuropil threads and neurofibrillary tangles, in the entorhinal cortex, hippocampus and other temporal regions, lead to an inability to store and retrieve memories of smell, and thereby to correctly identify odors (Attems et al., 2005). amygdala and hippocampal are also part of brain areas that typically show atrophy in AD, that is, AD signature regions, exhibiting large numbers of amyloid plaques and neurofibrillary tangles. We speculate that may account for the strong association between the two areas and olfactory function.

Therefore, we further analyzed the volumes of the amygdala and hippocampal subregions and found that the volumes of hippocampal subregions (HC_Body_L and HC_Tail_L) were significant difference in HC and MCI groups while the global hippocampal volumes were not. Hippocampal subfield volumes were found to be correlated with amyloid status (Aß42/40 negative versus positive) (Yildirim et al., 2023). Early autopsy studies also had shown that in Braak stages I and II, the hippocampus (CA1) is already affected, albeit mildly (Braak and Braak, 1991) and at later AD stages additional subnuclei were affected (Tsuchiya and Kosaka, 1990; Vogt et al., 1990). Although the reported findings are not fully consistent with our study, both suggest that analysis at the level of hippocampal subfields provides greater meaningful information than whole-hippocampal volumetry. Early researches on the hippocampal subregion could only be segmented into seven parts and the atlas only included right hippocampal subfields and the research reported the fimbria and fissure volumes were not significantly different between the amnestic mild cognitive impairment (aMCI) and the elderly control (EC) group (Hanseeuw et al., 2011). However, our study divided the two sides of the hippocampus into 14 parts respectively, and found that HC_Body_L and HC_Tail_L volumes were significantly smaller in the MCI than in HC group. The total volumes of brain regions may mask the early changes in the subregions, and the subregion measurements detect lesions earlier, or atrophy occurs earlier (Salman et al., 2024). Through Spearman partial correlation analysis between amygdala and hippocampal subregion volumes with olfactory function we found that compared with the total volumes of brain regions, more sub-region volumes have moderate-strength correlations with DT, IT, and TOS. Maybe the olfactory function and the volumes of subnuclei are good candidates as early biomarkers of preclinical AD.

## 5 Limitations

There are several potential limitations should be noted. First, in our study, only volume measurements were analyzed. Previous research clarified the atrophy rate of cortical thickness increases with progression of the disease from the preclinical to clinical stage. Besides, cortical thinning in subjects with HC converted to MCI was significantly faster than that in subjects with stable HC (Kulason et al., 2020; Henzen et al., 2025). Younes et al. found that the surface area of the entorhinal cortex also changes with age through differential morphological measurement indicators based on MRI (Younes et al., 2014). Therefore, we speculate that cortical thickness and surface area may also be imaging markers for the early diagnosis of AD continuum. In subsequent research, we will further analyze the relationship between structural indicators such as cortical thickness, cortical surface area, and cortical curvature and the AD continuum. Second, our research results are based on a limited sample size. Further studies in larger cohorts are needed to confirm these results and determine whether these indicators could be good candidates as early biomarkers for preclinical AD.

## 6 Conclusion

Our study identified the significance of olfactory function, especially IT, as well as the specific nuclei—HC_Body_L and HC_Tail_L of hippocampus, in the recognition of cognitive impairment by analyzing the hippocampus subnuclei volumes and the olfactory function among the three groups. Segmenting hippocampal subfields indeed allow improved sensitivity to MCI compared with total hippocampus volumetry. These findings are supported by both traditional neuropathological and neuroimaging studies. Therefore, we should highlight the studying of the relevance of the subnuclei and olfactory function in cognitive impairment.

## Data Availability

The datasets supporting the conclusions of this article are not readily available because the data also form part of an ongoing study. Requests to access the datasets should be directed to XH, 28804712@hebmu.edu.cn.
